# How to Prevent Chloroform Asphyxia

**Published:** 1876-03

**Authors:** 


How to Prevent Chloroform Asphyxia.—Dr. J. Fleiberg. (*Berl. Klin. Wochenschr.*, 1875, Sept.) His plan is to dislocate the inferior maxillary bone forward whenever any asphyxial symptoms make their appearance. The best way of producing this dislocation is to stand behind the reclining patient, put both thumbs behind the symphysis and the index fingers on the posterior edges of the rami of the bone, then grasp the maxilla firmly and drag it directly forward. If the patient be under the influence of the anæsthetic—and then only it is necessary to resort to this procedure—the condyle will move forward with a perceptible motion, and the entire bone is displaced. As soon as this is done the patient takes a long breath, and respiration proceeds without any further difficulty as long as the parts remain in the same position. The author has employed this procedure in more than a thousand instances, and has never failed to achieve the desired purpose, namely, the use of chloroform without any unpleasant complications. He believes that the root of the tongue and the epiglottis are dragged forward, and thereby the occlusion of the larynx and the consequent asphyxia, which is due to the weight of the tongue, are obviated.

The same method has been in constant use by Esmarch since 1866; and Langenbeck employed it constantly during the late Franco-German war, and never lost a case from the effects of chloroform.

